# Patient-Centred Outcomes after Totally Endoscopic Cardiac Surgery: One-Year Follow-Up

**DOI:** 10.3390/jcm12134406

**Published:** 2023-06-30

**Authors:** Jade Claessens, Pieter Goris, Alaaddin Yilmaz, Silke Van Genechten, Marithé Claes, Loren Packlé, Maud Pierson, Jeroen Vandenbrande, Abdullah Kaya, Björn Stessel

**Affiliations:** 1 Faculty of Medicine and Life Sciences, UHasselt—Hasselt University, Agoralaan, 3590 Diepenbeek, Belgium; abdullah.kaya@jessazh.be (A.K.); bjorn.stessel@jessazh.be (B.S.); 2 Department of Cardiothoracic Surgery, Jessa Hospital, Stadsomvaart 11, 3500 Hasselt, Belgium; alaaddin.yilmaz@jessazh.be (A.Y.); silke.vangenechten@jessazh.be (S.V.G.); loren.packle@jessazh.be (L.P.); 3 Department of Anesthesiology, Jessa Hospital, Stadsomvaart 11, 3500 Hasselt, Belgium; pieter.goris@jessazh.be (P.G.); marithe.claes@uhasselt.be (M.C.); piersonmaud@gmail.com (M.P.); jeroen.vandenbrande@jessazh.be (J.V.)

**Keywords:** quality of life, clinical outcomes, totally endoscopic cardiac surgery

## Abstract

Patient-centred outcomes have grown in popularity over recent years in surgical care research. These patient-centred outcomes can be measured through the health-related quality of life (HRQL) without professional interpretations. In May 2022, a study regarding patient-centred outcomes up to 90 days postoperatively was published. Fourteen days after surgery, the HRQL decreased and returned to baseline levels after 30 days. Next, the HRQL significantly improved 90 days postoperatively. However, this study only focuses on a short-term follow-up of the patients. Hence, this follow-up study aims to assess the HRQL one year after totally endoscopic cardiac surgery. At baseline, 14, 30, and 90 days, and one year after surgery, the HRQL was evaluated using a 36-item short form and 5-dimensional European QoL questionnaires (EQ-5D). Using the 36-item short form questionnaire, a physical and mental component score is calculated. Over the period of one year, this physical and mental component score and the EQ-5D index value significantly improve. According to the visual analogue scale of the EQ-5D, patients score their health significantly higher one year postoperatively. In conclusion, after endoscopic cardiac surgery, the HRQL is significantly improved 90 days postoperatively and remains high one year afterward.

## 1. Introduction

Research in the field of cardiac surgery predominantly focuses on reducing surgical trauma and improving the post-operative complications [1]. Recently, new totally endoscopic techniques were developed including totally endoscopic aortic valve replacement (TEAVR), endoscopic coronary artery bypass grafting (Endo-CABG), and mitral valve surgery through video-assisted thoracoscopic surgery (MVATS) [2,3,4,5,6]. Besides endoscopic AVR, transcatheter aortic valve implantation (TAVI) through the femoral artery has gained popularity and may be a viable alternative [7]. The aforementioned techniques are not yet state-of-the-art due to the technical difficulty and lack of clinical and patient-centred outcome data. Health-related quality of life (HRQL) is a measure of patient-centred outcomes that has gained importance in medical care. Through these outcome measures, the patient’s health status can be determined without the interpretation of a physician, allowing for a better understanding of how surgery affects the patient’s health [8]. The HRQL can be measured with questionnaires such as the short form 36 (SF-36) and the EuroQoL 5-dimension (EQ-5D). These questionnaires may meet the criteria for adequate surgical quality of recovery (QoR) measures [9]. There is a scarcity of prospective data on patient-centred outcomes, including HRQL and clinical results of these new totally endoscopic techniques. After our previously published study, which focused on patient-centred outcomes until 90 days after the surgery, the present study investigates the HRQL one year postoperatively [10]. In the previous study, the HRQL decreased over 14 days after surgery, then it returned to baseline levels at 30 days. After 90 days, the HRQL significantly improved. Our aforementioned study only focuses on a short-term follow-up of the patients. Hence, this follow-up study aims to assess the mid-term HRQL.

## 2. Materials and Methods

### 2.1. Study Design

This prospective longitudinal cohort study was authorized by Jessa Hospital’s local ethics committee (registration number B243201836445), Belgium, and submitted for inclusion to clinicaltrials.gov (NCT03902717). The Declaration of Helsinki was followed in this trial, and all participants gave their written approval through an informed consent form.

Between November 2019 and October 2020, all patients undergoing totally endoscopic cardiac surgery (TECS), TAVI, or standard open CABG were eligible to participate. The TECS procedures included TEAVR, Endo-CABG, MVATS, and concomitant endoscopic treatments. An age below 18 years, involvement in another experiment, prior heart surgery, and conversion to sternotomy during the surgery were exclusion criteria. However, patients planned for a CABG through sternotomy were included. The HRQL was assessed through the SF-36 and EQ-5D questionnaires orally or over the phone. The different time points were baseline (before the surgery), 14, 30, and 90 days, and one year after the surgery. The study design was previously described [10].

### 2.2. Surgical Techniques

The description of the surgical techniques has been published before [2,6]. All procedures were performed using endoscopic ports (5 mm), a utility port (2–3 cm), and peripheral cardiopulmonary bypass (CPB).

### 2.3. Quality of Life

The two questionnaires used to assess the HRQL are the SF-36 and EQ-5D questionnaires [11,12]. The SF-36 includes eight domains, from which a physical and mental component score (PCS and MCS) can be calculated. The means and standard deviations from a reference population of people with ischemic heart disease in Belgium were used to standardize the eight SF-36 scales [13]. All values above or below 50 are above or below the average of the reference population. The higher the PCS and MCS score, the higher the HRQL of the patient. The eight domains of the SF-36 include physical functioning, role limitations due to physical health, pain, general health, role limitations due to emotional problems, energy/fatigue, emotional well-being, and social functioning. An index value for the EQ-5D is calculated based on a crosswalk value set (the general population of the Netherlands, since no data are available for Belgium). This index value is calculated from five dimensions: mobility, self-care, daily activities, pain/discomfort, and anxiety. Additionally, patients rate their current health on a scale from 0 to 100 in the EQ visual analogue scale (VAS).

### 2.4. Clinical Follow-Up

The clinical outcomes investigated in this trial were readmission, reoperation, major adverse cardiac and cerebrovascular events (MACCE, including cardiac death, stroke, myocardial infarction, and target lesion revascularization), and neurological complications (cerebrovascular accident (CVA) and transient ischemic attack (TIA)). Target lesion revascularization is defined as a reintervention in the target vessel for CABG, and in the valve procedures, a reintervention of the valve involved. Additional clinical outcomes included graft failure, paravalvular leakage, permanent pacemaker (PPM) implantation, pericarditis, and 30-day and one-year mortality. Pericarditis is defined as the presence of two of the following criteria: a pericardial friction rub, retrosternal chest pain, ECG changes (wide-spread concave ST-segment elevation and PR-segment depression), and/or pericardial effusion [14].

### 2.5. Statistical Analysis

The Shapiro–Wilk test was used to determine whether the data were normally distributed. Continuous variables were displayed as the median and interquartile range (IQR). Numbers and associated percentages provided the description of categorical variables. For non-parametric data, a Kruskal–Wallis test was used to compare the various surgical techniques, and a chi-square test was applied for categorical variables. The differences between the baseline and postoperative HRQL were examined using the Friedman and Wilcoxon signed-rank tests. A Kaplan–Meier estimated survival plot was created for the 30-day and one-year mortality. A *p*-value smaller than or equal to 0.05 was considered significant. All statistical analyses were performed using R: A language and environment for statistical computing (R Core Team (2021), R Foundation for Statistical Computing, Vienna, Austria).

## 3. Results

**Figure 1:**
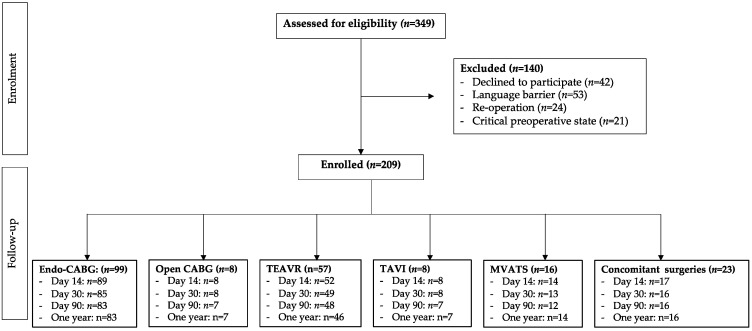
STROBE (strengthening the reporting of observational studies in epidemiology) flow chart of patient recruitment and follow-up. Endo-CABG: endoscopic coronary artery bypass grafting; TEAVR: totally endoscopic aortic valve replacement; TAVI: transcatheter aortic valve implantation; MVATS: mitral valve surgery through video-assisted thoracoscopic surgery.

Enrolment and follow-up numbers are displayed in a STROBE (strengthening the reporting of observational studies in epidemiology) flow chart in Figure 1.

### 3.1. Demographics

All subpopulations had similar outcomes except for the mean patient age in TEAVR versus TAVI ((73.0 (65.0–76.0) years versus 85.5 (77.8–87.8) years, *p* < 0.001) and the mean European system for cardiac operative risk evaluation (EuroSCORE) II in the open CABG group versus the Endo-CABG group, where the former was significantly higher (3.5 (1.9–4.6) versus 1.3 (0.9–2.2), *p* = 0.021). Demographics and medical history are described in Table 1 and in the previous manuscript [10].

### 3.2. Health-Related Quality of Life

**Figure 2:**
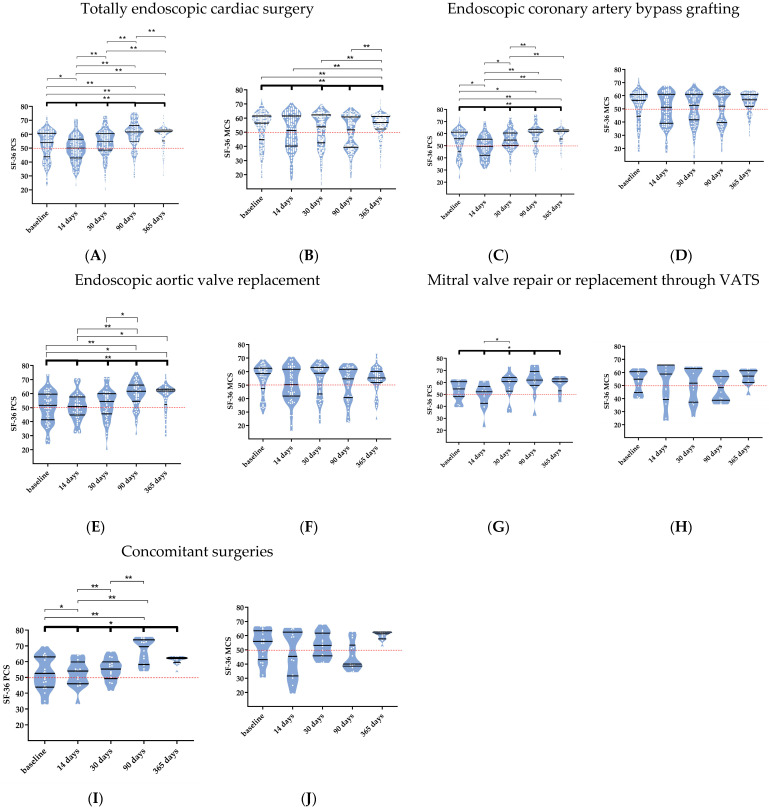
The physical and mental component scores (PCS and MCS) of the 36-item short form (SF-36) questionnaire after totally endoscopic cardiac surgery (**A**,**B**), endoscopic coronary artery bypass grafting (**C**,**D**), endoscopic aortic valve replacement (**E**,**F**), mitral valve repair or replacement through video-assisted thoracoscopic surgery (VATS) (**G**,**H**), and concomitant surgeries (**I**,**J**). Data are shown in truncated violin plots as medians and interquartile ranges. The reference line indicates the mean of a reference population (ischemic heart disease in Belgium, red line). The bold significance bar represents a Friedman analysis over the five time points. The other significance bars include post-hoc analyses using a pairwise Wilcoxon signed-rank test. Significance is marked as * *p* < 0.05 and ** *p* < 0.001.

Totally endoscopic cardiac surgery: In the overall TECS cohort, the physical component score (PCS) of the SF-36 significantly changed after surgery (*p* < 0.001, Figure 2A). One year after the surgery, the PCS did not further improve compared to 90 days (90 days: 61.6 (55.1–65.6), one year: 61.8 (55.7–63.0), *p* = 1, Figure 2A). In contrast to the PCS, the MCS did not change significantly over the whole study period after TECS (Figure 2B). When comparing one year with 14, 30, and 90 days, the MCS did significantly improve (one year: 57.1 (52.3*–*61.4); 14 days: 51.0 (39.8*–*61.4), *p* = 0.001; 30 days: 53.7 (42.7–62.1), *p* = 0.045; and 90 days: 51.7 (39.6–61.0), *p* = 0.001) but compared to baseline values, there was no significant improvement after one year (56.5 (45.3–61.5), *p* = 0.393, Figure 2B).

Physical functioning, pain, general health, emotional well-being, and social functioning all improved over time. On the other hand, role limitations due to physical and mental health and the patient’s energy/fatigue did not differ significantly over the five time points. Patients did not experience any role limitations due to mental health in any of the five time points. Additionally, an improvement was seen in role limitations due to physical health, pain, and social functioning between 90 days and one year (Figure S1).

**Figure 3:**
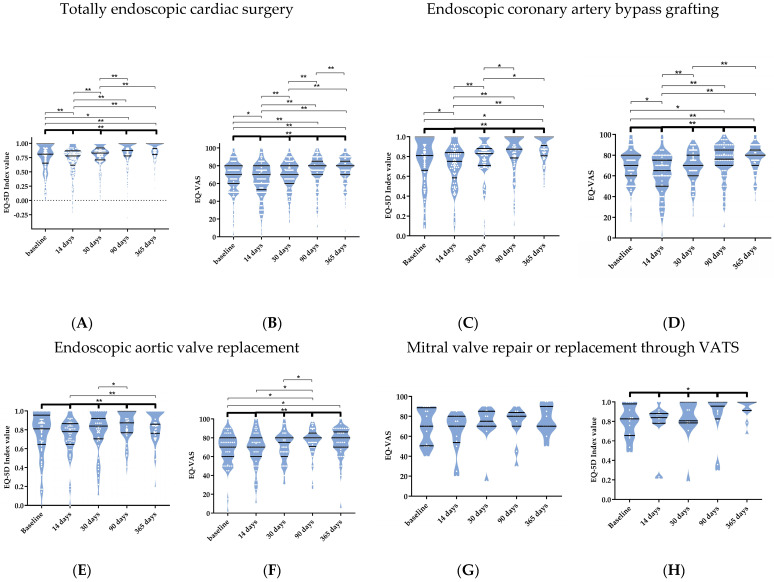

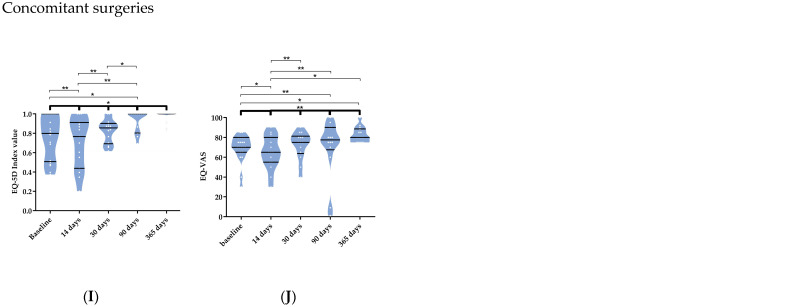
The index value and visual analogue scale (VAS) of the European quality-of-life 5-dimensional questionnaire (EQ-5D) after totally endoscopic cardiac surgery (**A**,**B**), endoscopic coronary artery bypass grafting (**C**,**D**), endoscopic aortic valve replacement (**E**,**F**), mitral valve repair or replacement through video-assisted thoracoscopic surgery (**G**,**H**), and concomitant surgeries (**I**,**J**). Data are shown in truncated violin plots as medians and interquartile ranges. The bold significance bar represents a Friedman analysis over the five time points. The other significance bars include post-hoc analyses using a pairwise Wilcoxon signed-rank test. Significance is marked as * *p* < 0.05 and ** *p* < 0.001.

In the overall TECS population, the EQ-5D questionnaire’s index score significantly increased over time (*p* < 0.001). Compared to baseline values, a significant decline was observed after 14 days (*p* < 0.001), followed by a return to baseline values at 30 days and a significant increase at 90 days (*p* = 0.015), and the index score remained at these levels after one year (0.9 (0.8;1.0), *p* < 0.001, Figure 3A). Additionally, the EQ-VAS and index score results were similar to the evolution of the index score in the overall TECS populations (Figure 3B).

**Figure 4:**
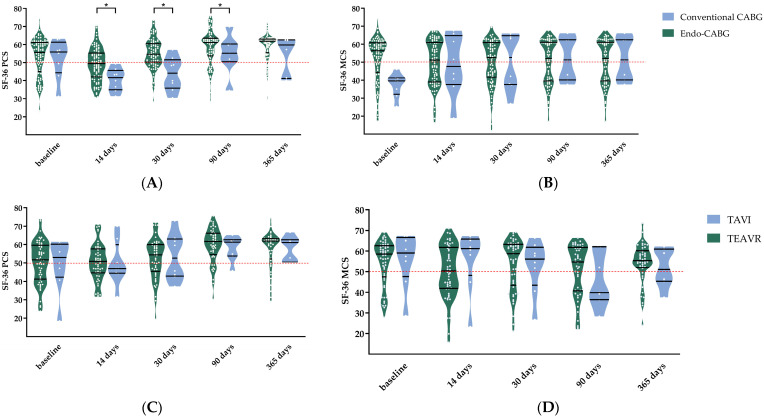

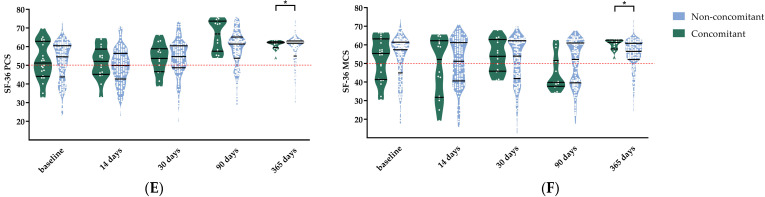
Comparison of the physical and mental component scores (PCS and MCS) of the short-form 36 (SF-36) questionnaire between endoscopic coronary artery bypass grafting (Endo-CABG) and open CABG (**A**,**B**), between totally endoscopic aortic valve replacement (TEAVR) and transcatheter aortic valve implantation (TAVI) (**C**,**D**), and between concomitant and non-concomitant (**E**,**F**). Data are shown in truncated violin plots as medians and interquartile ranges. The reference line indicates the mean of a reference population (ischemic heart disease in Belgium, red line). Significance is marked as * *p* < 0.05.

Coronary artery bypass grafting—The same significant improvements as the overall TECS population were seen in the PCS until one year after the surgery in the Endo-CABG patients (*p* < 0.001) with no further improvements between 90 days and one year (*p* = 1, Figure 2C). Following Endo-CABG, the MCS did not differ throughout the trial period (Figure 2D). Compared to open CABG, the PCS and MCS were not significantly different in the Endo-CABG group at different points in time (*p* = 0.396 and *p* = 0.128, respectively, Figure 4A,B).

In the Endo-CABG subpopulation, an improvement in physical functioning and role limitations due to physical health, pain, general health, and emotional well-being were observed over the five time points. The role limitations due to mental health and the patient’s energy/fatigue did not differ significantly over the five time points. In contrast to the other domains, social functioning levels decreased (Figure S2).

Moreover, both the EQ-5D index value and VAS significantly improved over the whole study period (*p* < 0.001 and *p* < 0.001) in the Endo-CABG subpopulation (Figure 3C,D). After open CABG, only the VAS significantly improved (*p* = 0.009). No significant difference can be observed in the EQ-5D index value and VAS when comparing open CABG to Endo-CABG.

Aortic valve replacement—Similar to the overall TECS and Endo-CABG subpopulations, significant improvements in PCS were seen until one year after the surgery in TEAVR (Figure 2E) (*p* < 0.001), with no improvements between 90 days and one year.

When comparing TEAVR and TAVI, no significant difference in the PCS and MCS was seen (*p* = 0.759 and *p* = 0.221, respectively, Figure 4C,D).

TEAVR patients significantly improved in physical functioning, pain levels, and general health. The other domains of the SF-36 did not change over the different time points. Moreover, social functioning is the only domain significantly better in the TEAVR subpopulation compared to TAVI (*p* = 0.031, Figure S3). Additionally, the EQ-VAS and index score results were similar in the TEAVR and TAVI subpopulations.

Totally endoscopic mitral valve surgery—Patients who underwent a MVATS encountered similar significant improvements in PCS (*p* = 0.028) with no improvements between 90 days and one year. The MCS did not improve nor diminish over the whole study period. Additionally, emotional well-being was the only SF-36 domain that improved (*p* = 0.030). All other levels were high at all different time points (Figure S4).

On the other hand, the EQ-5D index value significantly improved over the five time points (*p* = 0.009), while the VAS improvement was borderline not significant (*p* =0.059).

Concomitant surgeries—As in all previously described populations, the same significant improvements in PCS were seen until one year after the concomitant surgeries (*p* = 0.004), with no further enhancements between 90 days and one year. Additionally, isolated TECS procedures compared to concomitant TECS showed no significant difference between the two for the PCS and MCS (*p* = 0.273 and *p* = 0.716, respectively, Figure 3E,F).

Furthermore, the EQ-5D index value and VAS significantly improved during the study after concomitant surgeries without any difference with single procedures.

### 3.3. Clinical Follow-Up

The clinical outcomes during the one-year follow-up period are presented in Table 2. In the overall TECS population, 28 (14.7%) patients were readmitted to the hospital 23.5 (15.8–53.8) days after the surgery, of which 8.9% were within 30 days. The reason for these readmissions included pericarditis, pleural effusion, heart failure, atrial fibrillation, hypotension, unstable angina, syncope, in-stent stenosis, and bradycardia. A reoperation was needed in three (1.6%) patients, two for a tamponade, and one Endo-CABG patient also required a TEAVR after 240 days. Furthermore, a MACCE occurred in 11 (5.8%) patients. These included five (2.6%) patients suffering a cardiac death, one (0.5%) patient with a myocardial infarction, and three patients with a stroke. Lastly, two patients needed a target lesion revascularisation.

Paravalvular leakage was not diagnosed in the TEAVR, MVATS, or concomitant TECS subgroups. Within the TAVI subgroup, however, three patients developed a paravalvular leakage. A PPM implantation was performed in ten (5.2%) TECS patients. Pericarditis was diagnosed in 25 (13.1%) patients.

**Figure 5:**
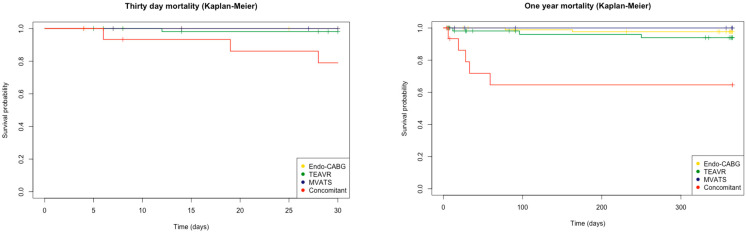
Estimated survival regarding the all-cause mortality (Kaplan–Meier) after 30 days and one year for endoscopic coronary artery bypass grafting (Endo-CABG), totally endoscopic aortic valve replacement (TEAVR), mitral valve repair or replacement through video-assisted thoracoscopic surgery (MVATS), and concomitant procedures.

In total, 10 (5.2%) TECS patients died during the one-year follow-up period. Half of them were during the hospital stay, and the other half were between 30 days and one year. The estimated survival after one year for Endo-CABG, conventional CABG, TEAVR, and concomitant procedures was 97.7%, 87.5%, 94.0%, and 64.4%, respectively (Figure 5). Within the TAVI and MVATS subpopulations, none of the patients died.

## 4. Discussion

### 4.1. Overall HRQL

This trial investigated the quality of life after totally endoscopic cardiac surgery after a one-year follow-up. The SF-36 PCS and MCS did not change between 90 days and one year during the follow-up period in all investigated populations. Additionally, in the TECS population, the EQ-5D index value and VAS did not further improve after 90 days. According to the first part of this study, 14 days after TECS, there is a reduction in overall HRQL. Then, 30 days after the procedure, these levels revert to baseline, and at 90 days, they significantly improve. In the procedure-specific groups, comparable outcomes were seen. This indicates that the process of recovery takes place during the first three months after surgery. The initial recovery phase is followed by a plateau phase of a good HRQL during the year following the surgery.

Our results are consistent with the results of Moscarelli et al [15]. In their study, a difference in EQ-5D between minimally invasive and conventional valve surgery was seen at three months, while at one year, no difference was observed. When comparing our quality-of-life results after TECS with the results of Moscarelli et al. after MICS, TECS resulted in a higher index value (MICS: 0.6 ± 0.2 and TECS: 0.912 (0.8;1.0)) and VAS (MICS: 72.4 ± 12.6 and TECS: 80 (70;85)) at 90 days after surgery [15]. The faster immediate recovery and faster re-establishment of the HRQL compared to conventional sternotomy, as described by Moscarelli et al., was also observed by Nasso et al., who investigated minimally invasive mitral valve repair [15,16]. The swift recovery process resulting in a high HRQL at three to six months after surgery observed in these two studies and our study may explain the lack of further improvement between 90 days and one year. A systematic review of studies investigating the HRQL measured with the SF-36 and EQ-5D after minimally invasive cardiac surgery indicated that, while both minimally invasive and traditional cardiac surgery may benefit the patient, those receiving minimally invasive cardiac surgery may recover more rapidly [17].

### 4.2. Clinical Follow-Up

At one year of follow-up after TECS, the clinical outcomes were promising. After Endo-CABG, a MACCE was observed in only 4.0%. Two studies investigating conventional CABG reported a MACCE rate of 5.1% and 7.7% [18,19]. In contrast, we did not observe a MACCE in the conventional CABG subpopulation of this study. This finding can be explained by the small sample size (*n* = 8) of this subgroup. This explanation also applies to the TAVI subgroup of this study (*n* = 8), in which no MACCE was seen also. Other studies reported a MACCE rate of 12% and 18.2% after TAVI and a MACCE rate of 15.8% and 17.6% after conventional open AVR [20,21]. In this trial, 7.0% of TEAVR patients experienced a MACCE. Moreover, the concomitant subpopulation had a higher occurrence of MACCE (13.0%) but aligned with the literature (11.5%) [22].

Furthermore, the readmission rate within 30 days was 8.9%, which is in line with the pooled 30-day readmission rate of 12.9% in the meta-analysis of Shawon et al. [23].

A PPM implantation was required in 5.2% of TECS patients. Especially after TEAVR, PPM implantation (12.3%) was higher compared to the literature (2–7%). The high PPM implantation rate observed in our study may be partially explained by a low threshold for pacemaker implantation at our hospital. In the literature, a PPM rate of 15.5% and 16.2% is reported after TAVI, which is still higher than in our TEAVR patients [20,21]. In this trial, the PPM rate of TAVI patients was also 12.5%. However, this depends on the type of valve prosthesis. In stented valves the PPM rate is significantly lower compared to sutureless valves. In this study, in 23.6% of patients, a sutureless valve was implanted.

The one-year mortality rate in the overall TECS population of this study was 5.2%, which is lower compared to other trials. After minimally invasive direct CABG, the reported mortality rate of 10.8% after a median follow-up of 11 months is five times higher than the observed mortality rate of 2.02% after Endo-CABG in our study [24]. Furthermore, after surgical AVR, the one-year mortality rate in other studies varies from 6.7% to 13.6%, compared to 5.3% in our study [20,25]. Regarding concomitant surgeries, the mortality rate (21.7%) observed in our study was higher than in other studies (8.4% and 11.0%). This observation may be partially due to the small sample size.

### 4.3. Limitations

In this study, the 30- and 90-day follow-up for most patients was during the COVID-19 pandemic, while the one-year follow-up was mainly afterwards. Presumably, the pandemic may have influenced our results regarding social and psychological functioning. For example, social functioning significantly improved after one year compared to 90 days. Several patients indicated that their social functioning was low due to the pandemic and not because of their health status.

Another limitation of this trial is the small sample size of the control groups (conventional CABG and TAVI). This is caused by the rapid switch to TECS in our centre. At the start of this trial, CABG was performed sporadically, and by the end of the trial, our centre became a 100% TECS centre. Regarding TAVI patients, the inclusion was more difficult due to the preoperative state of these patients. They were older, more fragile, and had more comorbidities, so they refused to participate in this trial more frequently. This created a huge selection bias between TEAVR and TAVI.

Lastly, the Hawthorne effect may always influence a trial with a questionnaire at multiple time points. Some patients may have reported higher HRQL due to the learning effect during the study period [26].

## 5. Conclusions

During the first three months after the surgery, the HRQL recovery process takes place. Following the initial phase of recovery, a plateau of a good HRQL is seen over the course of a year after surgery. The clinical follow-up during this period shows excellent morbidity and mortality rates. Our results indicate that implementation of TECS in the standard of care can have a positive effect on the HRQL and clinical outcomes after the surgery.

## Figures and Tables

**Table 1:** Demographics and medical history. Data are represented as *n* (%) and median (IQR).

	TECS (*n* = 193)	Endo-CABG (*n* = 99)	Open CABG (*n* = 8)	*p*-Value	TEAVR (*n* = 57)	TAVI (*n* = 8)	*p*-Value	MVATS (*n* = 16)	Concomitant (*n* = 23)
Age (years)	70.0 (62.0–77.0)	67.0 (61.5–73.5)	70.0 (68.0–75.5)	0.441	73.0 (65.0–76.0)	85.5 (77.8–87.8)	<0.001	72.5 (64.8–80.3)	75.0 (69.0–78.0)
BMI (kg/m^2^)	26.9 (25.0–29.8)	26.7 (25.1–29.6)	27.8 (22.7–29.4)	0.859	27.0 (24.0–30.9)	26.0 (24.5–26.9)	0.259	26.1 (22.9–28.1)	27.6 (26.0–30.9)
EuroSCORE II (%)	1.6 (1.0–2.6)	1.3 (0.9–2.2)	3.5 (1.9–4.6)	0.021	1.4 (1.0–2.3)	-	-	2.3 (1.9–3.4)	2.7 (2.0–6.0)
Gender (male)	146 (75.6)	86 (86.8)	6 (75.0)	0.352	33 (57.9)	5 (62.5)	0.805	13 (81.3)	15 (65.2)
Smoking				0.277			0.146		
- Active	42 (21.8)	24 (24.2)	4 (50.0)		11 (19.3)	0 (0.0)		5 (31.3)	3 (13.0)
- Stopped	38 (19.7)	22 (22.2)	1 (12.5)		8 (14.0)	3 (37.5)		1 (6.3)	7 (30.4)
DiM				0.840			0.480		
- Type I	4 (2.1)	3 (3.0)	0 (0.0)		1 (1.75)	0 (0)		0 (0)	0 (0)
- Type II	44 (22.8)	29 (29.3)	2 (25.0)		6 (10.5)	2 (25.0)		3 (18.8)	7 (30.4)
AHT	130 (67.4)	70 (70.7)	7 (87.5)	0.309	35 (61.4)	5 (62.5)	0.952	7 (43.8)	20 (87.0)
Profession				0.799			0.068		
- Independent contractor	11 (5.7)	7 (7.1)	0 (0.0)		4 (7.0)	0 (0.0)		0 (0.0)	0 (0.0)
- Employed	18 (9.3)	9 (9.1)	1 (12.5)		6 (10.5)	0 (0.0)		2 (12.5)	1 (4.4)
- Volunteer	0 (0.0)	0 (0.0)	0 (0.0)		0 (0.0)	0 (0.0)		0 (0.0)	0 (0.0)
- Unemployed	5 (2.6)	2 (2.0)	0 (0.0)		0 (0.0)	0 (0.0)		2 (12.5)	1 (4.4)
- Incapacity of work	11 (5.7)	8 (8.1)	0 (0.0)		1 (1.8)	0 (0.0)		1 (6.3)	1 (4.4)
- Retired	148 (76.7)	73 (73.7)	7 (87.5)		46 (80.7)	8 (100.0)		11 (68.8)	20 (87.0)
Education				0.696			0.067		
- Elementary	20 (10.4)	12 (12.1)	1 (12.5)		5 (8.8)	3 (37.5)		1 (6.3)	2 (8.7)
- Middle school	28 (14.5)	12 (12.1)	1 (12.5)		8 (14.0)	0 (0.0)		4 (25.0)	5 (21.7)
- High school	91 (47.1)	44 (44.4)	2 (25)		30 (52.6)	4 (50.0)		6 (37.5)	22 (47.8)
- Higher education	35 (18.1)	22 (22.2)	2 (25)		8 (14.0)	0 (0.0)		4 (25.0)	1 (4.4)
- University	17 (8.8)	8 (8.1)	2 (25.0)		5 (8.8)	0 (0.0)		1 (6.3)	4 (17.4)
- PhD	2 (1.0)	1 (1.0)	0 (0.0)		1 (1.8)	1 (12.5)		0 (0.0)	0 (0.0)

AHT: arterial hypertension; BMI: body mass index; DiM: diabetes mellitus; EuroSCORE II: European system for cardiac operative risk evaluation.

**Table 2:** Clinical outcomes during the follow-up period of one year in endoscopic coronary artery bypass grafting (Endo-CABG), conventional coronary artery bypass grafting (CABG), totally endoscopic aortic valve replacement (TEAVR), transcatheter aortic valve implantation (TAVI), mitral valve repair or replacement through video-assisted thoracoscopic surgery (MVATS), and concomitant procedures.

	TECS (*n* = 193)	Endo-CABG (*n* = 99)	CABG (*n* = 8)	TEAVR (*n* = 57)	TAVI (*n* = 8)	MVATS (*n* = 16)	Concomitant (*n* = 23)
Readmission	28 (14.7)	18 (18.2)	0	7 (12.3)	0	2 (12.5)	2 (8.7)
Reoperation	3 (1.6)	1 (1.0)	0	1 (1.8)	0	1 (6.3)	0
MACCE	11 (5.8)	4 (4.0)	0	4 (7.0)	0	0	3 (13.0)
- Cardiac death	5 (2.6)	0	0	2 (3.5)	0	0	3 (13.0)
- MI	1 (0.5)	0	0	1 (1.8)	0	0	0
- Stroke	3 (1.6)	2 (2.0)	0	1 (1.8)	0	0	0
- TLR	2 (1.1)	2 (2.0)	0	0	0	0	0
Neurological							
- CVA	2 (1.0)	1 (1.0)	0	1 (1.8)	0	0	0
- TIA	2 (1.0)	2 (2.0)	0	0	0	0	0
Graft failure	1 (0.9)	1 (1.0)	0	-	-	-	0
Paravalvular leakage	0	-	-	0	3 (37.5)	0	-
PPM implantation	10 (5.2)	2 (2.0)	0	7 (12.3)	1 (12.5)	1 (6.3)	0
Pericarditis	25 (13.1)	16 (16.2)	0	7 (12.3)	0	1 (6.3)	1 (4.3)
Mortality	10 (5.2)	2 (2.0)	1 (12.5)	3 (5.3)	0	0	5 (21.7)
- In hospital	5 (2.6)	0	0	1 (1.8)	0	0	4 (17.4)
- 30-day	0	0	0	0	0	0	0
- Follow-up	5 (2.6)	2 (2.0)	1 (12.5)	2 (3.5)	0	0	1 (4.4)

CVA: cerebrovascular accident; MACCE: major adverse cardiac and cerebrovascular event; MI: myocardial infarction; PPM: permanent pacemaker implantation; TIA: transient ischemic attack; TLR: target lesion revascularization.

## Data Availability

The data underlying this article will be shared on reasonable request by the corresponding author.

## Supplementary Materials

The following supporting information can be downloaded at https://www.mdpi.com/article/10.3390/jcm12134406/s1: Figure S1: Different domains of the Short Form 36 (SF-36) questionnaire after totally endoscopic cardiac surgery.; Figure S2: Different domains of the Short Form 36 (SF-36) questionnaire after totally endoscopic coronary artery bypass grafting; Figure S3: Different domains of the Short Form 36 (SF-36) questionnaire after totally endoscopic aortic valve replacement; Figure S4: Different domains of the Short Form 36 (SF-36) questionnaire after mitral valve repair or replacement through video assisted thoracoscopic surgery; Figure S5: Different domains of the Short Form 36 (SF-36) questionnaire after concomitant cardiac surgeries.
