# Supplementary material for: Patient-Centred Outcomes after Totally Endoscopic Cardiac Surgery: One-Year Follow-Up
Source: J Clin Med. 2023 Jun 30;12(13):4406. doi: 10.3390/jcm12134406 (PMC10342362; doi:10.3390/jcm12134406)
Supplement: Supplementary file 1 [file jcm-12-04406-s001.zip › Suplementary Figure S2.pdf]

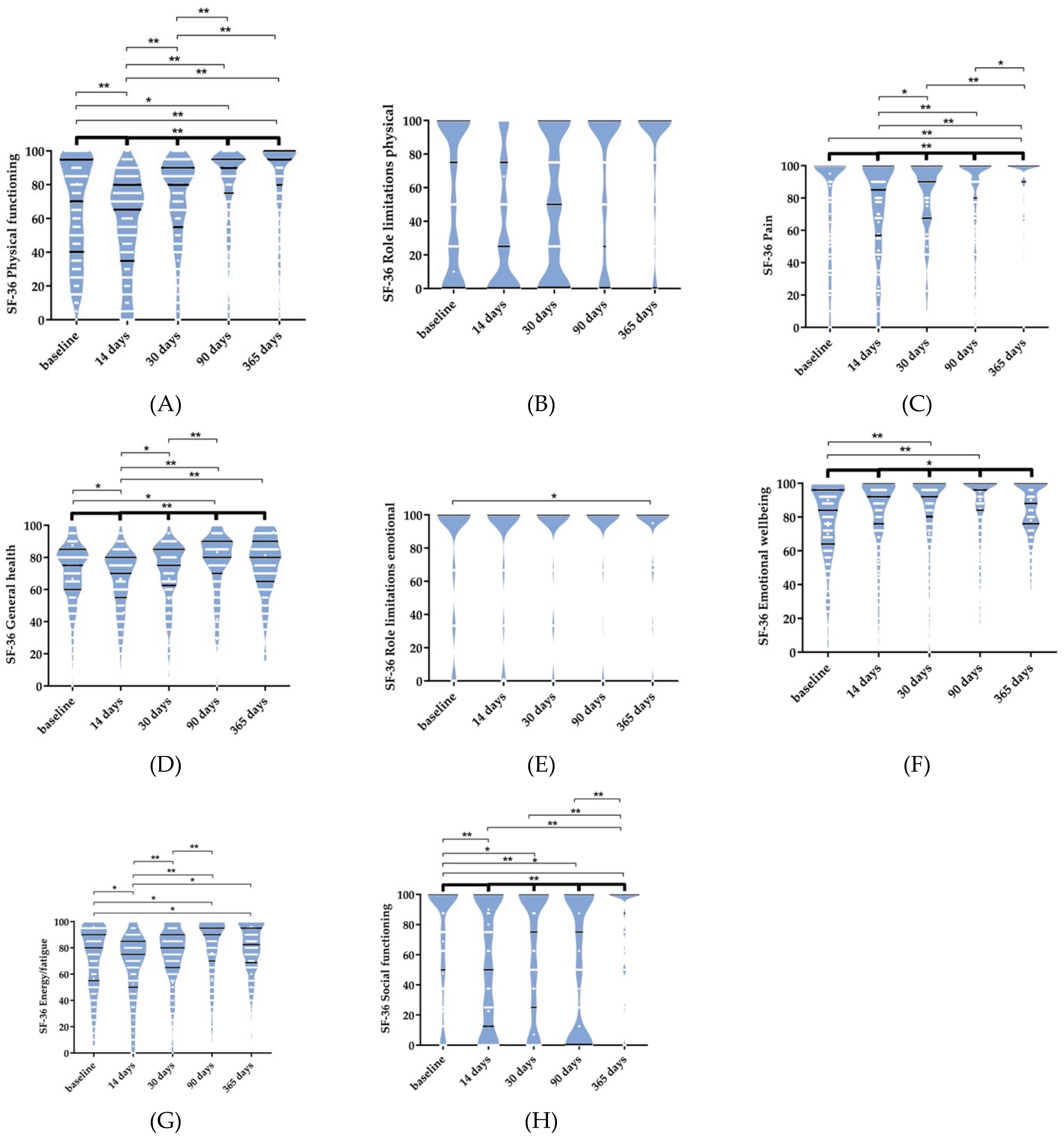

Supplementary Figure S2. Different domains of the Short Form 36 (SF-36) questionnaire after totally endoscopic coronary artery bypass grafting. These included physical functioning (**A**), role limitations physical (**B)**, pain (**C**), general health (**D**), role limitations emotional (**E),** emotional wellbeing (**F**), energy/fatigue (**G**) and social functioning (**H**). Data are shown as median and interquartile ranges. Significance is indicated as * $p < 0.05$; ** $p < 0.001$.
